# *Lactobacillus* Persisters Formation and Resuscitation

**DOI:** 10.4014/jmb.2312.12035

**Published:** 2024-01-29

**Authors:** Hyein Kim, Sejong Oh, Sooyeon Song

**Affiliations:** 1 Department of Animal Science, Jeonbuk National University, Jeonju 54896, Republic of Korea; 2 Division of Animal Science, Chonnam National University, Gwang-Ju 61186, Republic of Korea; 3 Department of Agricultural Convergence Technology, Jeonbuk National University, Jeonju 54896, Republic of Korea

**Keywords:** *Lactobacillus*, persistence, persister resuscitation

## Abstract

*Lactobacillus* is a commonly used probiotic, and many researchers have focused on its stress response to improve its functionality and survival. However, studies on persister cells, dormant cells that aid bacteria in surviving general stress, have focused on pathogenic bacteria that cause infection, not *Lactobacillus*. Thus, understanding *Lactobacillus* persister cells will provide essential clues for understanding how *Lactobacillus* survives and maintains its function under various environmental conditions. We treated *Lactobacillus* strains with various antibiotics to determine the conditions required for persister formation using kill curves and transmission electron microscopy. In addition, we observed the resuscitation patterns of persister cells using single-cell analysis. Our results show that *Lactobacillus* creates a small population of persister cells (0.0001–1% of the bacterial population) in response to beta-lactam antibiotics such as ampicillin and amoxicillin. Moreover, only around 0.5–1% of persister cells are heterogeneously resuscitated by adding fresh media; the characteristics are typical of persister cells. This study provides a method for forming and verifying the persistence of *Lactobacillus* and demonstrates that antibiotic-induced *Lactobacillus* persister cells show characteristics of dormancy, sensitivity of antibiotics, same as exponential cells, multi-drug tolerance, and resuscitation, which are characteristics of general persister cells. This study suggests that the mechanisms of formation and resuscitation may vary depending on the characteristics, such as the membrane structure of the bacterial species.

## Introduction

*Lactobacillus* spp. is a well-known probiotic strain [1]. *Lactiplantibacillus plantarum* is one of the most commonly found strains in fermented foods and has been well studied for its ability to influence gut health [2] and immune regulation [2-4] via antioxidant effects [5] and anticancer effects [6]. Processing probiotics in animal feed additives, pharmaceutical products, and starter cultures for dairy products is crucial to ensuring high survival rates [7]. Consequently, many researchers have focused on how *Lactobacillus* responds to cold, heat, acidity, osmosis, starvation, oxidative stress, and bile stress [8]. Understanding their stress response is vital to ensuring their viability and functionality in these diverse environments, including the gut [8, 9]. Like most bacteria, *Lactobacillus* survives these stresses by producing chaperone proteins [10, 11], regulating the composition of fatty acids in the membrane [12], producing osmoprotectants [13], and entering dormancy [9, 14].

During bacterial dormancy, the two primary states are viable but non-culturable (VBNC) [15] and persister cells [16]. The VBNC state refers to cells that remain non-revivable empty shells [17, 18]. In contrast, persister cells, which constitute a small fraction of the bacterial population (ranging from 0.00001–1%), enter a dormant “sleep mode” to withstand stress without undergoing genetic alterations [19]. Once the stress is removed, these persister cells can revive and regrow into normal cells [19].

To form bacterial persister cells, guanosine tetraphosphate (ppGpp) and cAMP, induced by stress, activate ribosomal factors such as raiA, RMF, and Hpf, which lead to the formation of persister cells. These persister cells subsequently inactivate ribosomes into 100S ribosomes [20]. When the stress is removed, these previously inactivated 100S ribosomes within persister cells can reactivate back into 70S ribosomes, a process facilitated by HflX, thus resuming normal protein synthesis [20, 21]. As the initial step towards reactivation, bacteria sense the presence of fresh media through chemotaxis and membrane receptors. Subsequently, they reduce the levels of the secondary messenger cAMP, which serves as a signal for rescuing the dimerized ribosomes [22].

Every species, including *Escherichia coli*, *Pseudomonas aeruginosa*, and *Staphylococcus aureus*, forms persister cells [23]. However, no studies have been conducted on whether *Lactobacillus* forms persister cells in response to stress. To our knowledge, this is the first report elucidating the formation and resuscitation of *Lactobacillus* persister cells.

## Materials and Methods

### Bacterial Strains and Growth

The *L. plantarum* 2305 (accession # OQ918274) stock strain was stored at -80°C. The strain was streaked on an MRS agar plate and incubated overnight at 37°C for culturing [24]. Then, single colonies were inoculated in fresh MRS broth and incubated overnight at 37°C. MRS broth cultured with *L. plantarum* 2305 was diluted 1:50 into MRS broth and incubated at 37°C until an optical density at 600 nm (OD_600_) reached 0.6 for exponential cells.

### Selection Antibiotics

To select antibiotics for inducing *L. plantarum* persister cell formation, *L. plantarum* exponential phase cells (200 μl) were loaded into a 96-well microplate, and eleven antibiotic stocks (10 mg/ml each) (amoxicillin, ampicillin, chloramphenicol, ciprofloxacin, erythromycin, gentamicin sulfate, kanamycin, penicillin G, rifampicin, tetracycline, and vancomycin) were added to a final concentration of 100 μg/ml (Table S1). As a control, antibiotic solvents (deionized water [DIW], dimethyl sulfoxide [DMSO], and 0.5 M NaOH) were tested at the same volume. The absorbance of the cell cultures with antibiotics and control solutions was measured using a microplate reader for 18 h. Cells (CFU/ml) treated with antibiotics for 18 h were washed with 0.85% NaCl and counted by dropping them onto MRS agar plates.

### Persister Cell Formation of *L. plantarum*

To determine the conditions (*e.g.*, antibiotic concentration and treatment time) required to form *L. plantarum* persister cells, various concentrations (300 and 400 μg/ml) of antibiotics were treated, and viable cells were counted. The treatment time was determined by counting viable cells treated with 400 μg/ml of ampicillin every 10 h for 74 h. *L. plantarum* persister cells incubated with 400 μg/mL ampicillin for 30 h exhibited a flat kill curve.

### Transmission Electron Microscopy (TEM) Imaging

Samples were prepared from *L. plantarum* exponential phase cells and treated with ampicillin for 20, 30, or 40 h. The samples were centrifuged, the supernatant was discarded, and the samples were washed two times using 2%paraformaldehyde and 2% glutaraldehyde in 0.05 M sodium cacodylate buffer, a primary fixing solution. The samples were stirred at 4°C for 1 h and stored overnight in a refrigerator at 4°C. After the supernatants were separated and removed, the samples were mixed with 0.05 M sodium cacodylate buffer and then stirred at 4°C for 3 min. The washing step was performed three times. Next, the supernatants were separated and removed, and 1%osmium tetroxide was added in 0.05 M sodium cacodylate buffer, mixed, and stirred at 4°C for 1.5 h.

The samples were washed two times with distilled water and centrifuged, and the supernatants were discarded. Subsequently, 0.05% uranyl acetate was added to each sample. The samples were incubated overnight at 4°C. Then, the sample was centrifuged, and the supernatant was removed. They were then sequentially dehydrated with 30, 40, 50, 70, 80, 90, and 100% ethanol. The mixture was then infused with 100% propylene oxide and stirred two times for 3 min. During the infusion process, the samples were treated with a mixture of propylene and resin. They were polymerized at 60°C for 48 h and cut using an ultramicrotome. Samples were observed under a Hitachi (H-7650) TEM.

### Minimum Inhibitory Concentration (MIC) Test

To measure the MIC of resuscitated *L. plantarum* persister cells, persister cells formed by ampicillin treatment are washed and then *L. plantarum* persister cells are diluted 1:50 into MRS broth and incubated at 37°C until reach at 0.6 of OD_600_. Resuscitated *L. plantarum* persister cells are diluted 1:1000 into MRS broth (2 ml) with 0, 0.2, 0.5, 1, and 2 μg/ml of ampicillin and incubated at 37°C. To measure the MIC of *L. plantarum* exponential cells, exponential cells are diluted 1:1000 into MRS broth (2 ml) with 0, 0.2, 0.5, 1, and 2 μg/ml of ampicillin and incubated at 37°C.

### Persister Resuscitation on Agarose Gel Pads

*L. plantarum* persister cells treated with 400 μg/ml ampicillin for 30 h were washed, and 5 μl was dropped onto a 1.2% agarose/MRS or 0.85% NaCl gel pad. A cover glass was put on the gel pad before observation under a microscope (Zeiss Axioscope5). The gel pad was incubated at 37°C and observed under a microscope at 0, 8, and 15 h after incubation.

### Persister Formation and Resuscitation of Another *Lactobacillus* Strain

*Limosilactobacillus fermentum* exponential phase cells were harvested in the same manner as *L. plantarum*. To select an antibiotic to form *L. fermentum* persister cells, *L. fermentum* exponential phase cells (200 μl) were loaded into 96-well microplates. Eleven antibiotics (10 mg/ml) were added to a final concentration of 100 μg/ml. As a control, antibiotic solvents were tested at the same volume. The absorbance of cell cultures with antibiotics and control solutions was measured using a microplate reader (BioTek Synergy HTX multi-mode reader) for 18 h. The cells (CFU/ml) were washed with 0.85% NaCl after treatment for 18 h and counted by dropping them onto an MRS agar plate.

The *L. fermentum* exponential cells were treated with amoxicillin, the selected antibiotic, at a concentration of 4 μg/ml for 60 h, and viable cells were counted every 10 h by dropping 10 μl of the culture on an MRS agar plate. To observe persister cell resuscitation, *L. fermentum* persister cells induced by 4 μg/ml amoxicillin for 30 h were prepared. Five microliters of persister cells were dropped onto a 1.2% MRS agarose gel pad. Persister cell resuscitation was observed using a microscope (Zeiss Axioscope5) at 0, 3, 5, 8, and 15 h after incubation.

### Statistical Analysis

All microbiological test were performed with two independent cultures and the averages of the data were reported. Each value shown is the mean ± standard error of the mean (error bar) from two independent cultures. Student’s *t*-tests were used to compare two groups (* indicates a *p* value < 0.05 and ** indicates a *p* value < 0.01)

## Results

### Ampicillin-Induced *L. plantarum* Persister Cell Formation

**Fig. 1:**
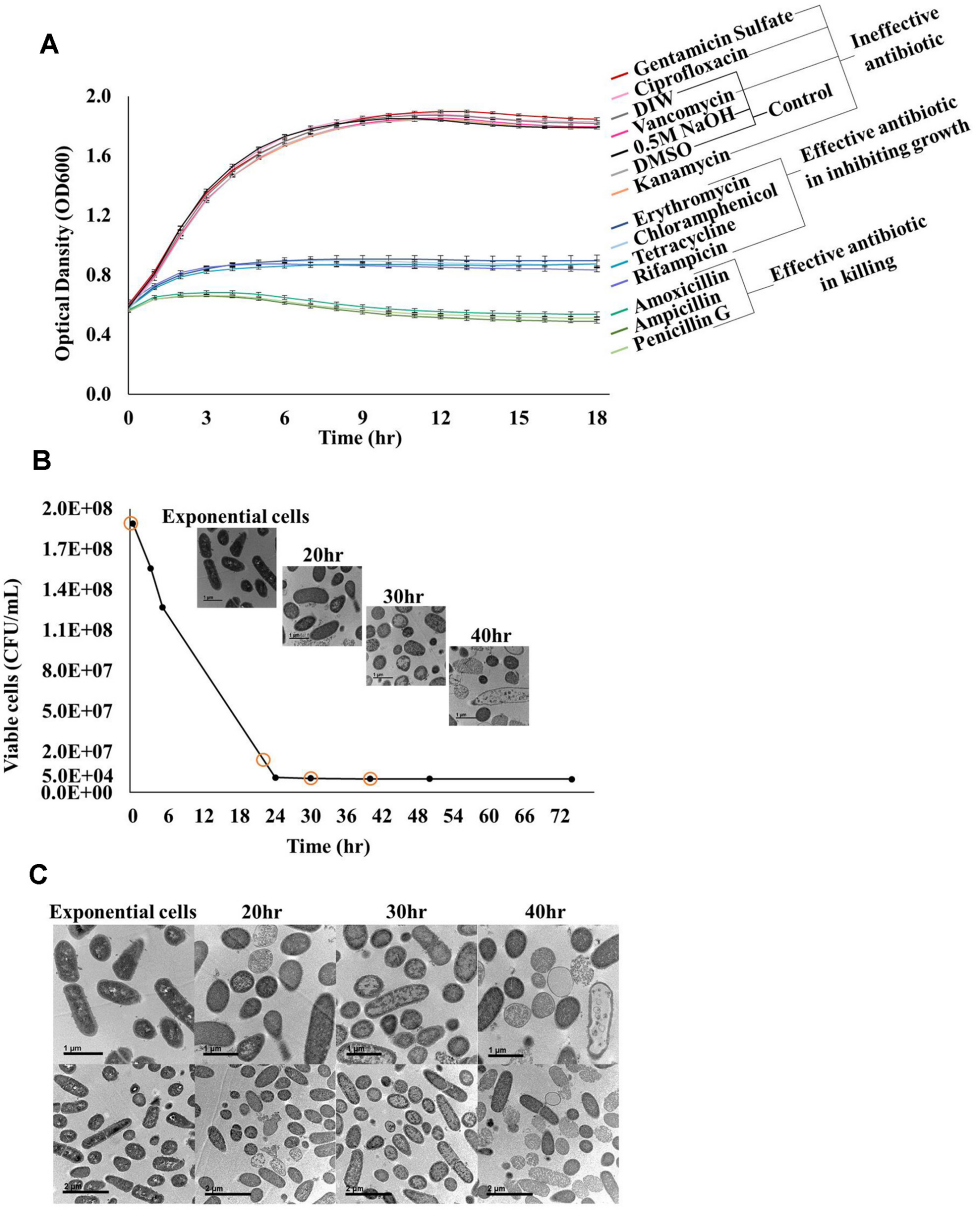
Formation of *L. plantarum* persister cells. (**A**) To select the antibiotics for *L. plantarum* persister formation, 11 antibiotics were administered to exponential-phase cells. According to the trend in the OD_600_ value of *L. plantarum*, the 11 antibiotics were classified into three groups: ineffective antibiotics, effective antibiotics for inhibiting growth, and effective antibiotics for killing. Solvents of 11 antibiotics, DIW, DMSO, and 0.5 M NaOH, were used as controls. Two independent cultures were used for this study. (**B**) Persister formation was determined by counting viable cells and obtaining TEM images. CFU/ml graphs of *L. plantarum* exponential cells treated with 400 μg/ml ampicillin for 74 h were drawn. The TEM pictures show exponential cells and cells treated with 400 μg/ml ampicillin for 20, 30, and 40 h. The scale bar indicates 1 μm. Two independent cultures were used for this study. (**C**) The TEM pictures show whether the exponential cells and cells treated with 400 μg/ml ampicillin for 20, 30, and 40 h are cytoplasmic-rich cells, persister cells, or VBNCs. The scale bar indicates 1 μm and 2 μm. Two independent cultures were used for this study.

We treated *L. plantarum* cells with eleven antibiotics (100 μg/ml) and selected an antibiotic that could effectively kill *L. plantarum* to form *L. plantarum* persister cells. The 11 antibiotics were divided into three categories: those that did not inhibit growth, those that inhibited growth, and those that killed *L. plantarum* cells. Amoxicillin, penicillin, ampicillin, and beta-lactam antibiotics that inhibit cell wall synthesis [25] were included in the group that kills *L. plantarum* cells (Fig. 1A, Table S2).

**Fig. 2:**
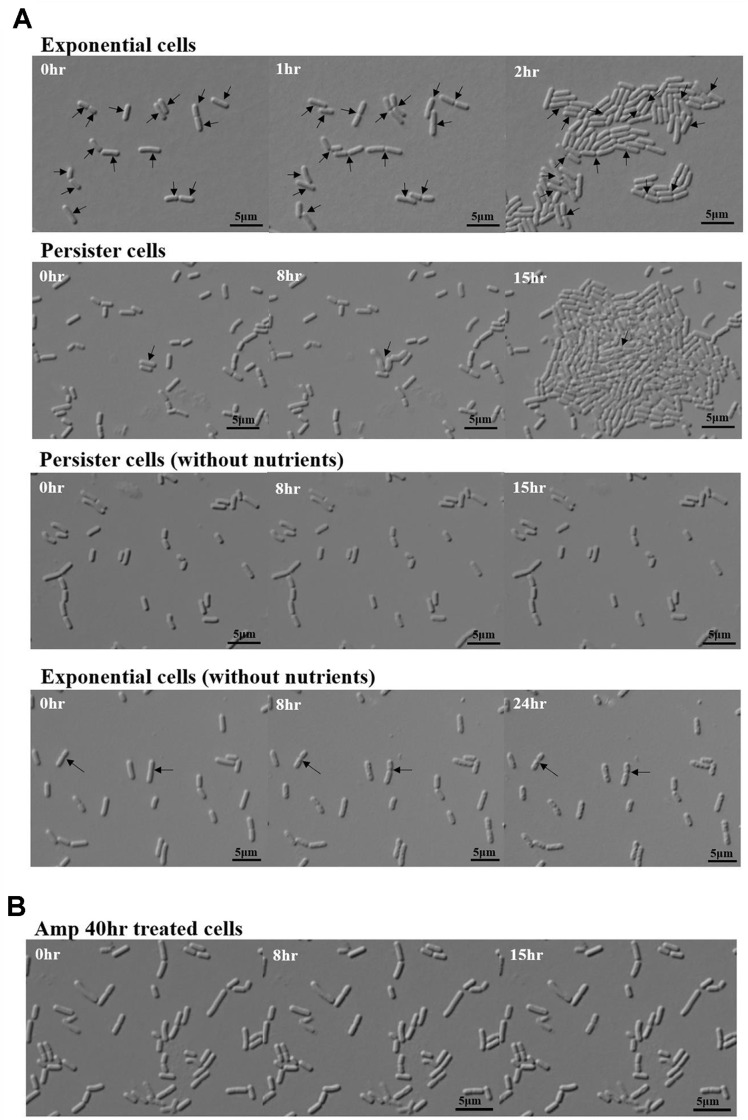
Resuscitation of *L. plantarum* persister cells. (**A**) All exponential cells imminently grow for 2 h at 37°C in MRS. Pictures show persistent cells resuscitating after incubation for 15 h at 37°C in MRS but not after 15 h at 37°C in 0.85% NaCl due to the lack of nutrients. In contrast, exponential cells grow at 0.85% NaCl. The black arrows indicate that the cells were resuscitated or grown. The scale bar indicates 5 μm. Two independent cultures were used for this study. (**B**) Persister cells treated with 400 μg/ml ampicillin for 40 h do not resuscitate after incubation for 15 h at 37°C in MRS. The scale bar indicates 5 μm. Two independent cultures were used for this study.

To determine the condition for forming persistent cells, exponential phase cells were treated with 400 μg/ml ampicillin, and the killing curve was checked (Fig. 1B, Table S3). A flat death curve, a characteristic of persister cells, was observed when cells were treated with ampicillin for more than 30 h (Fig. 1B). Additionally, to determine the appropriate treatment time for persister formation, the morphology of cells treated with ampicillin for 20, 30, and 40 h was confirmed using TEM (Fig. 1C). Kim *et al*. [17] observed *E. coli* persister cells and VBNC via TEM. They observed that healthy cells were rod-shaped with a condensed cytoplasm. In contrast, VBNC cells were empty shells without a cytoplasmic component. The persister cells were rod-shaped, but the cell wall was damaged or spherical. We observed that as the ampicillin treatment time increased, the rod-shaped cells of *L. plantarum* gradually became spherical, and the cytoplasmic content decreased (Fig. 1B and 1C). Exponential cells of *L. plantarum* had a condensed cytoplasm, and 20 h after ampicillin treatment, healthy, persister, and lysed cells were observed as a mixed population (Fig. 1B and 1C). In the kill curve phase, where cell count continues to decline, dying cells rather than persister cells are predominant. For accurate persister studies, it is crucial to observe cells in the phase where the kill curve becomes flat [26]. At 30 h, when the kill curve began to flatten, cells were observed to be rod-shaped, round, and had reduced cytoplasmic components or damaged cell walls. Most cells appeared VBNC 40 h after ampicillin treatment, with no cell contents or cell walls (Fig. 1C). The cells treated with antibiotics for 40 h were inoculated onto MRS agarose gel pads and observed for 15 h. Unlike persister cells, these cells could not be resuscitated (Fig. 2B). To comprehensively study only the persister cells of *L. plantarum*, except for resistance or tolerance, it is appropriate to study the persister cells formed by treatment with 400 μg/ml of ampicillin for 30 h.

Another characteristic of persister cell is that resuscitated persister cells have an antibiotic sensitivity same as exponential cells. We checked MIC of resuscitated persister cells and the MIC (0.5 μg/ml) for ampicillin is not changed compare to exponential cells (Table 1).

Furthermore, we checked the multi-drug tolerance of *L. plantarum* persister cells. To confirm that, we treated the antibiotics which killed *L. plantarum* such as amoxicillin, and penicillin. The persister cells (2.2E+0.5 CFU/ml) formed by ampicillin for 30 h survived by 1.1E+0.4 CFU/mL when treatment amoxicillin, and penicillin for 18 h respectively (Table 2).

To prove the dormancy state of persister cells, we observed the resuscitation of *L. plantarum* persister cells on the agarose gel which lacked the nutrients. The persister cells could not grow while the exponential cells grew on the 0.85% NaCl agarose gel (Fig. 2).

### *L. plantarum* Persister Cells Are Revived Heterogeneously

**Fig. 3:**
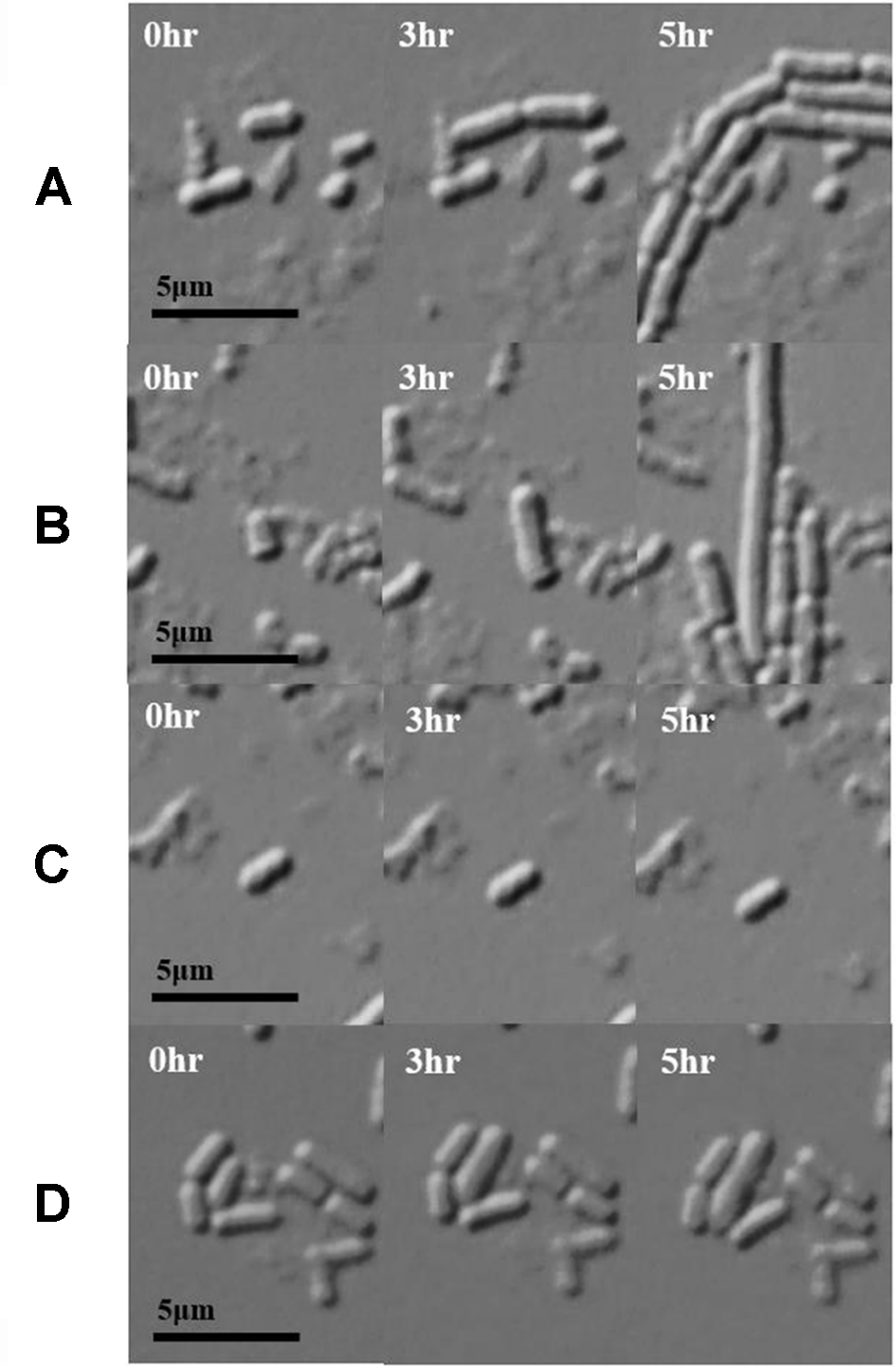
Resuscitation patterns. (**A**) Persister cells divide immediately and subsequently elongate. (**B**) Persister cells elongate and subsequently divide. (**C**) Persister cells remain in the same shape and do not resuscitate. (**D**) Persister cells elongate without division. All persister cells were rescued at 37°C for 5 h on an MRS agarose gel pad via microscopy.

To determine how *L. plantarum* persister cells resuscitate, ampicillin-induced persister cells were observed using single-cell analysis. For exponential-phase cells, all cells are immediately divided. In contrast, in *L. plantarum* persister cells, only 0.37 ± 0.07% of the cells were resuscitated for 8 h; even after 15 h, the other persister cells were not resuscitated (Fig. 2A, Table 3). Resuscitation of persister cells begins with the reactivation of ribosomes that were deactivated by stress [20, 21, 22, 27]. In *E. coli* persister cells, cell division occurs soon after the reactivation of ribosomes [22, 27]. Another characteristic of persister cells is their dormant state [28]. *L. plantarum* persister cells were revived on the MRS agarose gel but not on the 0.85% agarose gel. However, exponential cells of *L. plantarum* grew on 0.85% agarose gel (Fig. 2). This suggests that, similar to other persister cells, *L. plantarum* persister cells are in a dormant state (Fig. 2A). Kim *et al*. reported that *E. coli* persister cells do not survive without nutrients. In contrast, exponential cells divide even in the absence of nutrients [27]. This underscores the dormant nature of the persister cells. The resuscitation patterns of *L. plantarum* persister cells varied significantly (Fig. 3). These included cells that divided and subsequently elongated (Fig. 3A), cells that elongated and subsequently divided (Fig. 3B), cells that remained inactive without any changes (Fig. 3C), and cells that elongated without division (Fig. 3D). The waking patterns of *Lactobacillus* persister cells were observed when *E. coli* persister cells were resuscitated, and heterogeneous resuscitation occurred because each cell had a different ribosome activity [27]. However, in *Lactobacillus* persister cells, cells that elongated and then lysed were not observed. This likely results from differences in the cell wall composition of gram-positive and gram-negative bacteria. In the case of *E. coli* persister cells, cell lysis was induced even after washing with ampicillin since residual ampicillin affects reactivated cells [27]. However, the thicker cell walls of gram-positive bacteria like *Lactobacillus* might have prevented the lysis induced by any residual ampicillin. Elongation is the most commonly observed reactivation pattern in *Lactobacillus* persister cells. Elongation has been reported as a frequently occurring phenotype in response to antibiotic stress [29, 30]. However, the elongation observed after the removal of antibiotic stress has been observed in *E. coli* persister cells [27].

### Other Species of *Lactobacillus* Form and Regenerate Persister Cells

**Fig. 4:**
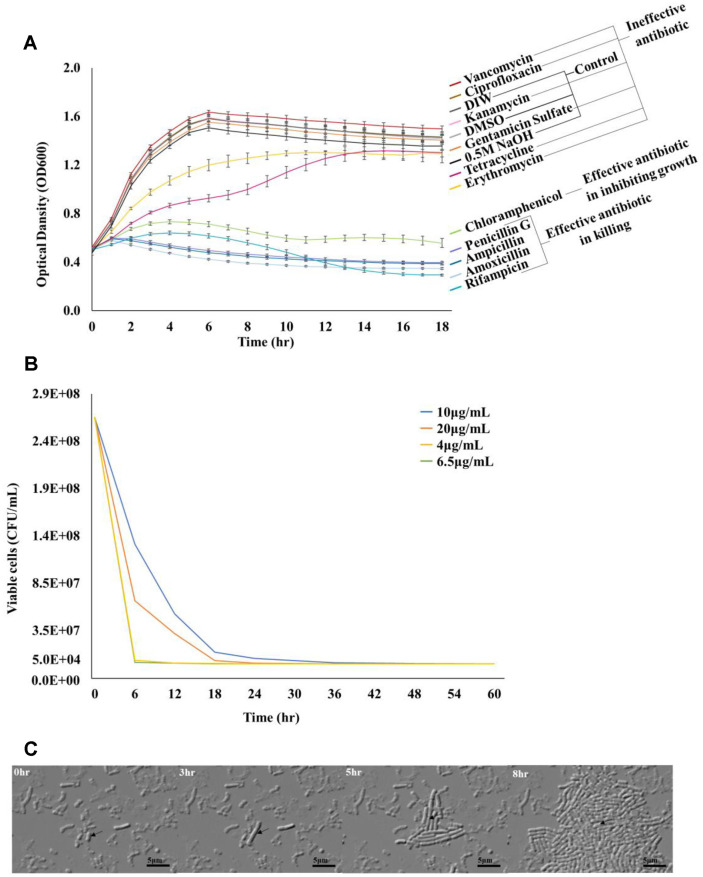
Formation and resuscitation of *L. fermentum* persister cells. (**A**) To select the antibiotics for *L. fermentum* persister formation, 11 antibiotics were administered to exponential-phase cells. According to the trend in the OD_600_ value of *L. fermentum*, the 11 antibiotics were classified into three groups: ineffective antibiotics, effective antibiotics for inhibiting growth, and effective antibiotics for killing. Solvents of 11 antibiotics, DIW, DMSO, and 0.5M NaOH, were used as controls. Two independent cultures were used for this study. (**B**) Persister formation was determined by counting the viable cells. CFU/ml graphs of *L. fermentum* exponential cells treated with four μg/ml amoxicillin for 60 h were drawn. The cells were treated with 4, 6.5, 10, and 20 μg/ml to determine the antibiotic concentration suitable for persister formation. Two independent cultures were used for this study. (**C**) *L. fermentum* persister cells resuscitate after 8 h of incubation at 37°C in MRS. The scale bar indicates 5 μm. Two independent cultures were used for this study.

To determine whether other *Lactobacillus* species formed and resuscitated persister cells, we generated *L. fermentum* persister cells and observed their resuscitation pattern (Fig. 4C). First, suitable antibiotics were selected to form persistent cells of *L. fermentum* using the same method used for generating *L. plantarum* persister cells. The antibiotics that killed *L. fermentum* were ampicillin, amoxicillin, penicillin G, and rifampicin (Fig. 4A), of which amoxicillin was the most effective (Table S4). Upon examining the kill curve for *L. fermentum* by treating the cells with various concentrations (4, 6.5, 10, and 20 μg/ml) of amoxicillin for 60 h, the curve became flat after 30 h of treatment with a concentration of 4 μg/ml (32 MIC) (Fig. 4B, Table S5).

We observed the resuscitation of *L. fermentum* persister cells formed by amoxicillin (4 μg/ml) on an MRS agarose gel pad via microscopy. *L. fermentum* persister cells (1.02 ± 0.05%) were resuscitated for 8 h, and the resuscitation was initiated after 3 h (Fig. 4C). We observed elongation of the waking pattern when the *L. fermentum* persister cells were resuscitated (Fig. 4C).

Our results showed that *Lactobacillus* forms a small population of persister cells (0.0001–1%) in response to beta-lactam antibiotics such as ampicillin and amoxicillin. We confirmed the persister cells are dormant, did not change their MIC compare to exponential cells, and have multi-antibiotics tolerance. In their dormant state, these persister cells can be resuscitated by removing stress and adding fresh media, characteristics that are typical of persister cells. Furthermore, we observed that the rate of persister formation, resuscitation, and speed of resuscitation varied among the strains.

## Discussion

Our study proposes a method to form *Lactobacillus* persister cells. *L. plantarum* was treated with ampicillin, and *L. fermentum* was treated with amoxicillin for more than 30 h to form persister cells. These persister cells were confirmed using a kill curve, TEM, MIC test, Multi-drug tolerance test, and single-cell analyses (Figs. 1, 2, and 4, Tables 1 and 2). The ubiquitous formation mechanism of persister cells depends on stress-induced inactivation of ribosomes, which ultimately leads to dormancy [20]. A previous study reported that *Lactobacillus* ribosomes are inactivated when exposed to stress [31]. This indicates that *Lactobacillus* is present in the persister cells because of the consequent inactivation of ribosomes.

We demonstrated that *Lactobacillus* persister cells comprised less than 0.1% of the total cell population and had the typical characteristics of persister cells: being dormant having multi-antibiotics tolerance and restarting heterogeneously when nutrients were supplied with the sensitivity of antibiotics same as exponential cells (Figs. 2 and 3, Tables 1 and 2) [27].

In this study, the resuscitation percentage of *Lactobacillus* persister cells was 0.5–1%, which was significantly lower than the 50% resuscitation rate of *E. coli* persister cells induced by ampicillin [27]. When an *E. coli* persister cell reactivates, it first senses nutrients using chemotaxis and phosphotransferase membrane proteins; then, a decrease in secondary messenger cAMP reactivates the inactivated ribosomes, causing the persister cell to move toward nutrients [22]. In this process, *E. coli* uses their flagella, but the presence of flagella in *Lactobacillus* is not a common characteristic [32], and few flagellated *Lactobacillus* spp. are observed [33]. Therefore, *Lactobacillus* persister cells use a different mechanism than *E. coli* persister cells to detect and move toward nutrients, which may explain why the resuscitation rate of *Lactobacillus* is low compared to *E. coli* persister resuscitation.

Another reason for the low resuscitation rate of *Lactobacillus* persister cells may be the differences in the cell walls of gram-positive and gram-negative bacteria. *Lactobacillus* has a thick and simple layer of peptidoglycan. In contrast, gram-negative *E. coli* has cell walls composed of two membranes (outer and cytoplasmic membranes), a periplasmic space, and a layer of peptidoglycan between the two membranes [34]. This means that gram-positive bacteria may be more vulnerable to stress due to their simple cell walls than gram-negative bacteria, which have double cell walls. For example, it has been reported that the cell wall of *Lactobacillus* becomes thinner owing to acid stress [35]. Our results showed that, when treated with antibiotics for 40 h, cells with only cell-shaped contents and without cell walls were observed (Fig. 1C). In addition, cells that died after elongation were observed in ampicillin-induced *E. coli* persister cells when they were resuscitated [27], whereas such a pattern could not be observed when *Lactobacillus* persister cells were resuscitated (Fig. 3); this is explained by the difference in the cell wall composition. Ampicillin, which remained in the periplasmic space between the double membranes of *E. coli* cells, induced lysis when the cells were reactivated. However, in gram-positive bacteria, there was no space where ampicillin could remain.

## Conclusion

Here, we demonstrate that *Lactobacillus* persister cells are formed by antibiotic stress and resuscitated by removing the stress. The *Lactobacillus* persister cells have typical persister cell characteristics, such as dormancy, antibiotics sensitivity of resuscitated persister cells, multi-antibiotics tolerance, and heterogeneous resuscitation after removing stress. This study provides a basis for identifying the formation and resuscitation mechanisms of *Lactobacillus* persister cells and sheds light on new stress response mechanisms in *Lactobacillus*. Furthermore, studying the persistence mechanisms of new species will provide important perspectives on antibiotic resistance.

## Supplemental Materials

Supplementary data for this paper are available on-line only at http://jmb.or.kr.



## Figures and Tables

**Table 1:** MIC measurement of exponential cell and resuscitated persister cell for ampicillin.

Ampicillin (μg/ml)	0	0.2	0.5	1
Exponential cell (OD_600_)	2.11	0.17	0.00	0.00
Resuscitated persister cell (OD_600_)	2.04	0.02	0.00	0.00

Exponential cell and resuscitated persister cell with the same OD_600_ value diluted 1:1000 into MRS broth with 0, 0.2, 0.5, 1 μg/ml of ampicillin and incubated for 24 h at 37°C. MIC was determined by measuring OD_600_. Two independent cultures were used.

**Table 2:** Viable cells of *L. plantarum* persister cell treated antibiotics (penicillin G, amoxicillin) at 12 h.

Antibiotics	Number	Persister cells induced ampicillin (CFU/ml)	Viable cells (CFU/ml)	StDev
Amoxicillin	#1	2.67E+05	1.08E+04	1.06E+02
	#2	1.77E+05	1.05E+04	
Penicillin G	#1	2.67E+05	1.13E+04	2.21E+02
	#2	1.77E+05	1.11E+04	

*L. plantarum* persister cells were treated with 100 μg/ml amoxicillin, and penicillin for 12 h at 37°C in MRS. Two independent cultures were used. StDev represents the standard deviation between the first and second cultures of the two independent cultures. A student’s *t*-test was used to compare persister cells vs. persister cells with antibiotics.

**Table 3:** Single *Lactobacillus* persister cell waking for 8 h.

Strain	Number	Total cells	Total waking cells	% waking
*L. plantarum* 2305	#1	319.9	1	0.37 ± 0.07
	#2	242	0.8	
*L. fermentum* 762G	#1	198.8	2	1.02 ± 0.05
	#2	176	1.75	

Single persister cells were observed using light microscopy (Zeiss Axioscope 5). The number indicates that results are the observations from two independent experiments. The total number of *L. plantarum* 2305 and *L. fermentum* 762G persister cells that wake on MRS gel pads is shown after 8 h at 37°C. Total waking cells indicates the number of dividing or elongating cells. The microscope images are shown in Figs. 2 and 4.
