# Supplementary material for: Lactobacillus Persisters Formation and Resuscitation
Source: J Microbiol Biotechnol. 2024 Jan 29;34(4):854–62. doi: 10.4014/jmb.2312.12035 (PMC11091699; doi:10.4014/jmb.2312.12035)
Supplement: Supplementary file 1 [file jmb-34-4-854-supple.pdf]

J. Microbiol. Biotechnol.
https://doi.org/10.4014/jmb.2312.12035

Supplementary Tables

Lactobacillus Persisters Formation and Resuscitation

Hyein Kim[1], Sejong Oh[2]*, and Sooyeon Song[1,3]*

Departments of Animal Science[1] and Agricultural Convergence Technology[3], Jeonbuk National University, 587 Baekje-Daero, Deojin-Gu, Jeonju-Si, Jellabuk-Do 54896, Republic of Korea

[2]Division of Animal Science, Chonnam National University, 77 Yongbong-Ro, Buk-Gu, Gwang-Ju 61186, Republic of Korea

*For correspondence. E-mail: songsy@jbnu.ac.kr

Table S1. Antibiotics list used in this study.

Antibiotics	*L. plnatnarum* MIC (μg/mL)	*L. fermentum* MIC (μg/mL)	Corporation	Chemical formula
**Amoxicillin**	0.5 [1]	0.125 [5]	MB Cell	$C_{16}H_{21}N_3O_7S$
**Ampicillin**	8 [2]	0.5 [4]	MB Cell	$C_{16}H_{18}N_3NaO_4S$
**Chloramphenicol**	4 [3]	8 [4]	Sigma	$C_{11}H_{12}Cl_2N_2O_5$
**Ciprofloxacin**	128 [3]	8 [4]	Sigma	$C_{17}H_{18}FN_3O_3$
**Erythromycin**	32 [4]	>64 [4]	MB Cell	$C_{37}H_{67}NO_{13}$
**Gentamicin Sulfate**	128 [3]	128 [4]	MB Cell	$C_{21}H_{45}N_5O_{11}S$
**Kanamycin**	512 [3]	>256 [4]	MB Cell	$C_{18}H_{38}N_4O_{15}S$
**Penicillin G**	4 [1]	0.5 [4]	MB Cell	$C_{16}H_{17}KN_2O_4S$
**Rifampicin**	8 [6]	8 [6]	MB Cell	$C_{43}H_{58}N_4O_{12}$
**Tetracycline**	>128 [4]	4 [4]	MB Cell	$C_{22}H_{25}ClN_2O_8$
**Vancomycin**	128 [3]	>256 [4]	MB Cell	$C_{66}H_{76}Cl_3N_9O_{24}$

Table S2. Viable cells of *L. plantarum* treated with effective antibiotics at 18h.

In drawing a kill curve experiment using a microplate reader, *L. plantarum* 2305 was treated with 100 µg/mL antibiotics (amoxicillin, ampicillin, chloramphenicol, ciprofloxacin, erythromycin, gentamicin sulfate, kanamycin, penicillin G, rifampicin, tetracycline, vancomycin) for 18 h at 37°C in MRS. Viable cells (CFU/mL) indicate the number of viable cells in two independent cultures treated with effective antibiotics (amoxicillin, ampicillin and penicillin G) recovered from the microplate. The number indicates that results are the observations from two independent experiments. StDev represents the standard deviation between the first and second cultures of the two independent cultures. A student's t-test was used to compare cells without antibiotics vs. with antibiotics (** indicates a p value < 0.01). The kill curve graph is shown in **Figure 1A.**

Antibiotics	Number	Viable cells (CFU/mL)	Average	StDev
**Amoxicillin**	**#1**	2.53E+06	2.53E+06**	2.29E+05
	**#2**	2.36E+06		
**Ampicillin**	**#1**	1.39E+06	1.32E+06**	1.05E+05
	**#2**	1.25E+06		
**Penicillin G**	**#1**	6.58E+05	6.48E+05**	1.53E+04
	**#2**	6.37E+05		

Table S3. Viable cells of *L. plantarum* treated ampicillin (400µg/mL) for 74h.

L. plantarum 2305 was treated with 400µg/mL ampicillin for 74 h at 37°C in MRS. Two independent cultures were used. StDev represents the standard deviation between the first and second cultures of the two independent cultures, and % survival is the percentage of viable cells at each time to cells (CFU/mL) at 0 h.

Number	Time (hours)	Viable cells (CFU/ mL)	StDev	% Survival
**#1**	**0**	1.90E+08	1.41E+06	100 ± 0
**#2**		1.88E+08		
**#1**	**3**	1.63E+08	1.06E+07	82.3 ± 4.4
**#2**		1.48E+08		
**#1**	**5**	1.42E+08	2.05E+07	67.5 ± 8.3
**#2**		1.13E+08		
**#1**	**24**	1.60E+06	5.89E+05	0.63 ± 0.20
**#2**		7.67E+05		
**#1**	**30**	2.67E+05	6.36E+04	0.12 ± 0.02
**#2**		1.77E+05		
**#1**	**40**	1.10E+05	4.87E+04	0.04 ± 0.02
**#2**		4.11E+04		
**#1**	**50**	1.80E+04	8.49E+02	0.009 ±0.0004
**#2**		1.68E+04		
**#1**	**74**	1.13E+03	3.54E+01	0.0006 ± 0.00002
**#2**		1.08E+03		

Table S4. Viable cells of *L. fermentum* treated with effective antibiotics at 18h.

In drawing a kill curve experiment using a microplate reader, *L. fermentum* 762G was treated with 10 μg/mL antibiotics (amoxicillin, ampicillin, chloramphenicol, ciprofloxacin, erythromycin, gentamicin sulfate, kanamycin, penicillin G, rifampicin, tetracycline, vancomycin) for 18 h at 37°C in MRS. Viable cells (CFU/mL) indicate the number of viable cells in two independent cultures treated with effective antibiotics (amoxicillin, ampicillin, penicillin G and rifampicin) recovered from the microplate. The number indicates that results are the observations from two independent experiments. StDev represents the standard deviation between the first and second cultures of the two independent cultures. A student's t-test was used to compare cells without antibiotics vs. with antibiotics (** indicates a p value < 0.05). The kill curve graph is shown in **Figure 4A.**

Antibiotics	Number	Viable cells (CFU/mL)	Average	StDev
**Amoxicillin**	**#1**	1.06E+06	1.07E+06*	4.71E+03
	**#2**	1.07E+06		
**Ampicillin**	**#1**	4.93E+06	6.17E+06*	1.74E+06
	**#2**	7.40E+06		
**Penicillin G**	**#1**	9.90E+06	1.09E+07*	1.39E+06
	**#2**	1.19E+07		
**Rifampicin**	**#1**	7.57E+07	7.15E+07*	5.89E+06
	**#2**	6.73E+07		

Table S5. Viable cells of *L. fermentum* treated amoxicillin (4µg/mL) for 60 h.

L. fermentum 762G was treated with 4µg/mL ampicillin for 60 h at 37°C in MRS. Two independent cultures were used. StDev represents the standard deviation between the first and second cultures of the two independent cultures, and % survival is the percentage of viable cells at each time to cells (CFU/mL) at 0 h.

Number	Time (hours)	Viable cells (CFU/ mL)	StDev	% Survival
#1	0	2.42E+08	2.56E+07	100 ± 0
#2		2.79E+08		
#1	6	4.00+06	2.83E+05	1.47 ± 0.2
#2		3.60E+06		
#1	12	6.20E+05	1.06E+05	0.27 ± 0.01
#2		7.7E+05		
#1	18	2.17E+05	1.27E+04	0.08 ± 0.009
#2		1.99E+05		
#1	24	1.18E+05	1.11E+04	0.04 ± 0.005
#2		1.03E+05		
#1	36	8.18E+04	1.06E+03	0.03 ± 0.002
#2		8.03E+04		
#1	48	5.43E+04	4.89E+03	0.02 ± 0.003
#2		4.73E+04		
#1	60	3.43E+04	1.65E+03	0.01 ± 0.001
#2		3.20E+04		

References

1. Delgado, Susana, Ana Belén Flórez, Baltasar Mayo. 2005. Antibiotic susceptibility of *Lactobacillus* and *Bifidobacterium* species from the human gastrointestinal tract. *Curr. Microbiol*. **50**: 202-207.

2. Pulido RP, Omar NB, Lucas R, Abriouel H, Cañamero MM, Gálvez A. 2005. Resistance to antimicrobial agents in lactobacilli isolated from caper fermentations. *Antonie Van Leeuwenhoek* **88**: 277-281.

3. Rojo-Bezares B, Sáenz Y, Poeta P, Zarazaga M, Ruiz-Larrea F, Torres C. 2006. Assessment of antibiotic susceptibility within lactic acid bacteria strains isolated from wine. *Int. J. Food. Microbiol.* **111**: 234-240.

4. Nawaz M, Wang J, Zhou A, Ma C, Wu X, Moore JE, Xu J, *et al.* 2011. Characterization and transfer of antibiotic resistance in lactic acid bacteria from fermented food products. *Curr. Microbiol*. **62**: 1081-1089.

5. Al-Ahmad A, Ameen H, Pelz K, Karygianni L, Wittmer A, Anderson AC, *et al.* 2014. Antibiotic resistance and capacity for biofilm formation of different bacteria isolated from endodontic infections associated with root-filled teeth. *J. Endod*. **40**: 223-230.

6. Manzoor A, Ul-Haq I, Baig S, Qazi JI, Seratlic S. 2016. Efficacy of locally isolated lactic acid bacteria against antibiotic-resistant uropathogens. *Jundishapur. J. Microbiol*. **9**: e18952.
